# Word Repetition and Isolation are Intertwined in Children’s Early Language Experiences

**DOI:** 10.1162/opmi_a_00172

**Published:** 2024-11-22

**Authors:** Mira L. Nencheva, Jessica F. Schwab, Casey Lew-Williams, Caitlin M. Fausey

**Affiliations:** Department of Psychology, Princeton University; Center for Teaching and Learning, Stanford University; Department of Psychology, University of Oregon

**Keywords:** infant-directed speech, isolation, repetition, word-learning

## Abstract

Infants experience language in the context of a dynamic environment in which many cues co-occur. However, experimenters often reduce language input to individual cues *a priori* without considering how children themselves may experience incoming information, leading to potentially inaccurate conclusions about how learning works outside of the lab. Here, we examined the shared temporal dynamics of two historically separated cues that are thought to support word learning: repetition of the same word in nearby utterances, and isolation of individual word tokens (i.e., single-word utterances). In a large database of North American English, we found that word repetition and isolation frequently co-occurred in children’s natural language experiences, and the extent to which they did so was linked to words’ earlier age of acquisition. This investigation emphasizes children’s experiences in time as a way to understand the learning cues in the language environment, which may help researchers build learning theories that are grounded in real-world structure.

## INTRODUCTION

Infants’ language experiences are embedded in a dynamic environment in which multiple cues simultaneously unfold over time. Infants need to keep track of which sounds, syllables, and words precede and follow each other, while also noting when caregivers emphasize a word, gesture to an object, or use emotion. The temporal structure of infants’ language experiences has important consequences for how infants find meaningful units from the speech stream and construct meaning (Graf Estes et al., 2007; Lany & Saffran, 2010; Lew-Williams et al., 2011; Saffran & Kirkham, 2018; Schwab & Lew-Williams, 2016). However, researchers typically focus on the temporal structure of one cue at a time. For example, they investigate word repetition as an independently useful cue, or isolated words (i.e., single-word utterances with pauses at the edges) as an independently useful cue. Is this siloing of learning cues the right way to pursue testing hypotheses about early learning, or are researchers imposing biases on what they think the world is like for the infant? The current investigation demonstrates the importance of considering the structure of language across multiple concurrent cues in order to gain a richer understanding of how infants learn from the complexities of incoming speech. We do this by focusing on two long-studied cues in children’s early speech input – isolated words and repetition – and we show that they are intertwined.

### The Multiple Features of Infant-Directed Speech

To explain early language learning, scientists have often focused on the speech cues available to children. Infant-directed speech (IDS) is a mode of speech used in many cultures and communities when interacting with infants and young children (Hilton et al., 2022). IDS represents a collection of cues, including melodic features, such as its higher and more variable pitch, exaggerated prosody, and vocal characteristics, as well as structural features, such as simpler and shorter utterances, and more repetition of sounds, syllables, words, and phrases, compared to adult-directed speech (Brodsky et al., 2007; Cameron-Faulkner et al., 2003; Fernald et al., 1989; Küntay & Slobin, 1996; Piazza et al., 2017; Schwab et al., 2018; Soderstrom, 2007; Tal et al., 2024). Together, these features of IDS have been proposed to enhance infants’ attention and learning across ages spanning the first two years of life (Cusack & Carlyon, 2003; Graf Estes & Hurley, 2013; Küntay & Slobin, 1996; Ma et al., 2011; ManyBabies Consortium, 2020; Nencheva & Lew-Williams, 2022; Nencheva et al., 2021; Thiessen et al., 2005). The hallmark features of IDS have been studied together in prior studies, for example, by seeing which cues or combinations of cues predict learnability (Braginsky et al., 2016; Brent & Siskind, 2001; Swingley & Humphrey, 2018). Although these studies include the overall frequency of multiple features (and, on occasion, their interactions), each feature has been defined and treated separately, without consideration for how it co-occurs with other features in time.

We do not necessarily know if language development researchers have defined input cues in a way that captures children’s experiences across time, and prior work may be subject to scientists’ historical biases in carving up the landscape of input into component features. What would happen if we were to reconstrue the cues available to young learners based on how children encounter these features? As one window into this question, we focused on two established features of IDS, each of which is thought to be an important cue in early language learning: caregivers’ repetition of the same word in nearby utterances (Brodsky et al., 2007; Hills, 2013; Schwab et al., 2018), and their use of single-word (isolated) utterances (Aslin et al., 1996; Brent & Siskind, 2001; Fernald & Morikawa, 1993; Siskind, 1996), both of which are more common in IDS than ADS. We examined the extent to which these cues are independent vs. intertwined features of IDS in children’s real-world experiences.

Single-word utterances containing isolated instances of a word are common in IDS, comprising at least 9% of English-speaking caregivers’ utterances (Aslin et al., 1996; Brent & Siskind, 2001; Fernald & Morikawa, 1993; Siskind, 1996; Van de Weijer, 1999), with some variability across different words (Swingley & Humphrey, 2018). These short, simplified linguistic utterances are even more common when caregivers respond to their child’s speech-like vocalizations (Elmlinger et al., 2019). For instance, a child may vocalize toward a toy truck, and their caregiver may respond by saying the word “truck” in isolation. Isolated words likely connect to word production and learning through a variety of cognitive processes. By definition, isolated words have pauses at their edges, and have been shown to facilitate young children’s detection of word boundaries within a novel syllable stream (Lew-Williams et al., 2011). Isolated words have also been shown to support word recognition in the first year of life (Gout et al., 2004; Houston & Jusczyk, 2000; Jusczyk & Aslin, 1995) and word learning in the second year of life (Brent & Siskind, 2001; Swingley & Humphrey, 2018). They have additionally been shown to enhance infants’ long-term memory of words (Karaman & Hay, 2018; Keren-Portnoy et al., 2019). Correlational evidence suggests that words encountered frequently in isolation were among the first words that children learned, and were also words that children used more often and embedded in their own single-word utterances (Brent & Siskind, 2001; Ninio, 1992). Isolated words may even be a better predictor of later child production than word frequency (Brent & Siskind, 2001).

Another key feature of IDS is caregivers’ repetition of sounds, syllables, words, and phrases across relatively short periods of time (Brodsky et al., 2007; Cameron-Faulkner et al., 2003; Hills, 2013; Hoff-Ginsberg, 1985, 1986; Küntay & Slobin, 1996; Lester et al., 2022; Newport & Gleitman, 1977; Schwab et al., 2018; Snow, 1972; Tal et al., 2024). Corpus analyses suggest that between 27% and 58% of caregiver utterances contain some degree of partial repetition (Brent & Siskind, 2001; Onnis et al., 2008b). This pattern may emerge naturally in communication between caregivers and children. Caregivers are likely to continue talking about the same toy while the child is playing with it, commonly described as discourse continuity (Chang & Deák, 2019; Frank et al., 2013; Horowitz & Frank, 2015; Luong et al., 2013; Schwab & Lew-Williams, 2020). This sometimes results in repetitions of that label in a way that supports word learning. For example, a caregiver in the Providence corpus (Demuth et al., 2006) said the following utterances in close temporal proximity: *“How about you count your monkeys,” “Put your monkeys in a hat,” “How many monkeys do you have,” “Put the monkeys in the barrel.”* Word repetition may provide a temporally salient cue, one that affords an opportunity to encode or reactivate the representation of a word form multiple times (Hintzman, 1976) within a short timescale (such as seconds or minutes). Indeed, lab-based experimental studies with 2- to 3-year-olds suggest that repetition supports early language learning (Horst et al., 2011; Newman et al., 2016; Schwab & Lew-Williams, 2016, 2020). The amount of word repetition in child-directed speech decreases over developmental time, suggesting that caregivers are sensitive to their child’s growing communicative capacities and adjust their production of useful structures accordingly (Kaye, 1980; McRoberts et al., 2009; Schwab et al., 2018).

Developmental scientists have usually investigated isolation and repetition as independent cues, with the exception of one acknowledgment of their temporal co-occurrence by Brent and Siskind (2001). It is easy to imagine that these two cues may co-occur as caregivers and children talk about the same toy over several utterances (resulting in repetition), and the caregiver provides an isolated form of the word in response to the child’s vocalizations. As just one illustrative example of this from the Providence corpus (Demuth et al., 2006), a caregiver said, “*Where’s your fingers?*”, “*Fingers*,” “*Can you say fingers?*”. To the extent that isolated words and repetition co-occur in close temporal proximity in IDS, could the power of these cues for learning and memory arise from their mutual operation rather than only as independent supports for language learning?

### Moving Beyond Predefined Individual Features of Infant-Directed Speech

A large theoretical body of work has argued for the merit of taking a non-reductionist approach to language (Behrens, 2009; MacWhinney, 2001; Marchman, 1997). Shifting the focus away from the so-called individual features of language may reveal that the rich and multifaceted structure of real-world language usage is more than the sum of individual features, as currently operationalized. Beyond the domain of language, there is evidence that infants may especially benefit from multiple concurrent cues. For example, in a visual prediction task, Yurovsky, Boyer, et al. (2013) found that a single strongly predictive cue was most beneficial for adults (and those benefits were diluted when additional cues were introduced), whereas infants benefited the most from having access to multiple predictive cues. This suggests that what matters for infant learning may not be the individual cues present, but rather the moments when multiple cues occur together. Further, the features that matter for learning may depend on children’s past language experiences, and may, in fact, be a combination of multiple cues that scholars typically study separately. In many domains, there is evidence that caregivers frequently use more than one communicative cue at a time (e.g., combining speech and gesture; Kosie & Lew-Williams, 2024), and the presence of multiple overlapping cues has been shown to support infant attention (Bahrick & Lickliter, 2000; Flom & Bahrick, 2007; Suarez-Rivera et al., 2019) and learning (Booth et al., 2008; Coffey et al., 2024; Frank et al., 2009; Lew-Williams et al., 2019).

In the case of word isolation and repetition, as stated above, we are not the first to take note of the possibility that they travel together in input. Across 14 recording sessions of speech to 9- to 15-month-old English-learning infants, Brent and Siskind (2001) found that mothers used a total of 63 distinct isolated word types, on average. Among these, 27% occurred two or more times within 30 seconds. They briefly noted that “mothers tend to use a variety of word types in isolation and tend to repeat a number of those word types in close temporal proximity” (p. B37). This finding – that isolation and repetition may co-occur in IDS –was intriguing and suggestive, but it seems that it did not take root in developmental scientists’ thinking. When one looks at published studies on these cues, isolation, and repetition are often confounded, even in lab-based experimental investigations that claim that isolation alone benefits word learning (Lew-Williams et al., 2011). Therefore, although there is indirect evidence that isolation and repetition likely co-occur and work together to support word learning, no research has systematically examined these two cues in tandem or estimated their real-world co-occurrence at scale.

## CURRENT INVESTIGATION

The current investigation probes (1) how frequently isolation and repetition co-occur in child-directed input, defined here as caregiver speech input available to the child in free-play caregiver-child interactions, and (2) whether the co-occurrence of these two features is a useful way to understand the cues that support children’s early language learning. Specifically, we predicted that isolated words would be disproportionately repeated in surrounding utterances, and that the more children encounter a given word in these *isolation-repetition clusters*, the earlier the word would be learned. To test these predictions, we computed the age of acquisition of nouns and verbs in a large database of over 9,000 North American English toddler vocabularies (Wordbank; Braginsky, 2023; Frank et al., 2017, 2021). We then analyzed corpora of transcribed North American English caregiver-child interactions to quantify when and with what frequency isolation and repetition were used by caregivers for each noun. This allowed us to assess whether repetition and isolation tend to co-occur in time, and whether caregivers’ use of a word in isolation-repetition clusters is predictive of earlier age of acquisition. Our study highlights the importance of understanding how features of natural communication are frequently intertwined in time and cannot be fully understood by artificially isolating components of child-experienced dynamics.

## STUDY 1: ARE ISOLATED NOUNS AND VERBS ALSO REPEATED?

In Study 1, we investigated whether isolation and repetition are likely to co-occur in children’s language input. We did so in two ways. First, we compared how close isolated vs. multiword instances of the same noun or verb are to repetitions of the same word. We hypothesized that if isolation and repetition are linked, then isolated instances of nouns and verbs will also be repeated more closely in time than multiword instances of the same word. Second, we tested whether caregivers who use a lot of words in isolation are also more likely to use more repetition. Preregistered analyses focused on nouns, in line with previous experimental work on isolation and repetition (Schwab & Lew-Williams, 2016). We also carried out exploratory replications of the same analyses with verbs.

### Method

We analyzed publicly available transcriptions of caregiver-child interactions (see Dataset). Preregistered hypotheses and analysis plans are available on aspredicted.org (https://aspredicted.org/2ZZ_G2F; Nencheva et al., 2021). Numerical data and analysis code are available on Open Science Framework (https://osf.io/gs628/?view_only=1a515aa5e0d6460bafb00efa4919202c; Nencheva et al., 2024).

#### Dataset.

We sampled caregiver utterances from 28 corpora (a total of 235 speakers) that captured interactions between English-speaking caregivers and their 9- to 30-months old children (430,504 total utterances; Child Language Data Exchange System, MacWhinney, 2000a, 2000b; including datasets from Bates et al., 1991; Bernstein, 1982; Bliss, 1988; Bloom, 1970; Bloom et al., 1974; Braunwald, 1971; Brown, 1973; Clark, 1978; Demetras, 1989; Demuth et al., 2006; Gleason, 1980; Higginson, 1985; Kuczaj, 1977; McCune, 1995; MacWhinney, 2000b; MacWhinney & Snow, 1990; Ninio et al., 1994; Rollins, 2003; Sachs, 1983; Soderstrom et al., 2008; Suppes, 1974; Tardif et al., 1999; Valian, 1991; Van Houten, 1986; Warren-Leubecker, 1982; Weist et al., 2009). The dataset was skewed toward the older end of the age range (see Supplementary Figure 1). This dataset is structured into different corpora, each of which consists of multiple transcribed caregiver-child interactions (transcripts). Each speaker within a corpus has a unique identifier that is consistent across transcripts (if the corpus contains multiple interactions with the same speaker, as is the case for some longitudinal corpora). On average, transcripts contained 284.9 caregiver utterances, and 203.1 child utterances. There were on average 4.2 words per caregiver utterance, and 2.0 words per child utterance.

#### Utterances Containing Nouns.

Within these corpora, we identified caregiver utterances that contained early-learned nouns (including plural forms) selected from the MacArthur-Bates Communicative Development Inventory (MB-CDI) Words & Sentences form (Fenson, 2007). From the 365 nouns on the MB-CDI, 265 nouns appeared in the transcripts. Thus, we used the childesr package (Braginsky et al., 2022, version 2.3) to identify the 107,478 utterances in which caregivers said one of these nouns (265 distinct noun types). For the first analysis, we included a subset of 261 nouns (98%) that were used at least twice in a transcript. This allowed us to compute the distance to the closest repetition for each noun instance. For all other analyses, we included all 265 nouns. This sampling scheme permits joint analyses of isolated and repeated noun use, along with how such patterns may matter for learning.

#### Utterances Containing Verbs.

For exploratory analyses, we also identified caregiver utterances that contained early-learned action verbs (including their inflections) from the MB-CDI Words & Sentences form (Fenson, 2007). We did not include auxiliary verbs, because they rarely occur in isolation. All 103 action verbs on the MB-CDI appeared in the transcripts (in 156,661 utterances). Since all verbs appeared in transcripts in which the verb was used at least twice, the full set of verbs was included in all analyses.

#### Measures

##### Utterance-Level Measures.

**Figure 1.**
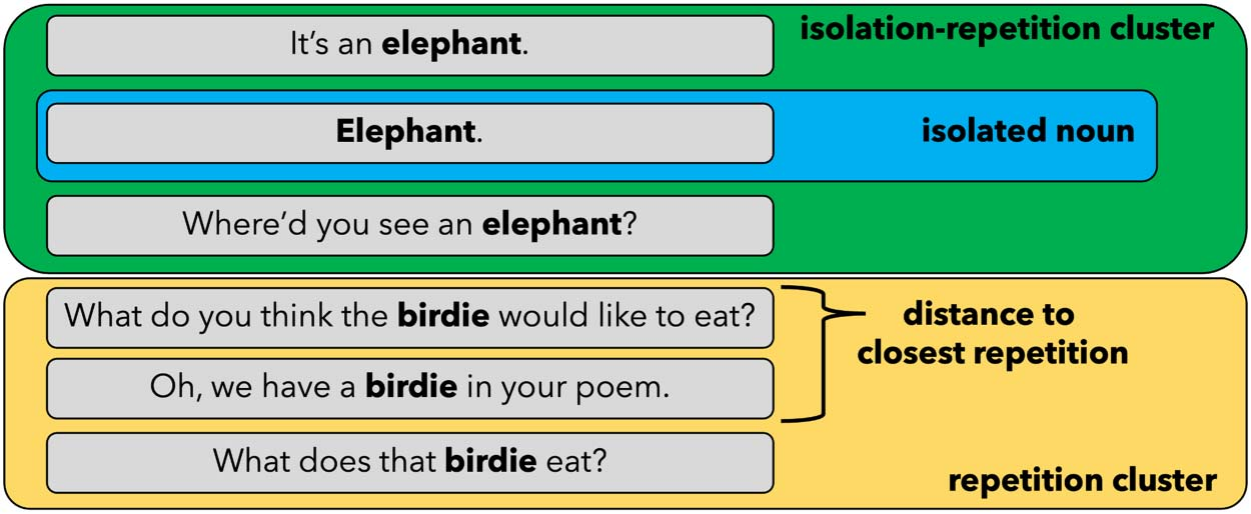
Examples from the Valian corpus (Valian, 1991) of an isolation-repetition cluster (circled in green), an isolated noun (circled in blue), and a repetition cluster (circled in yellow). The distance to the closest repetition of a noun is shown for one of the utterances.

Utterances were tagged as *isolated* if they consisted of a single word (the target word), or as a *multi-word* utterance if they contained one or more words in addition to the target word (see Figure 1). This resulted in 5,760 isolated nouns (5.4%) and 101,718 multi-word noun instances (out of all nouns in the analyzed dataset), and 3,724 isolated verbs (2.3%) and 152,937 multi-word verb instances (out of all verbs in the analyzed dataset). Next, for each utterance containing a target word, we quantified its proximity to the closest instance of the same word before or after the utterance. This was computed as the number of caregiver utterances between the two word instances in the transcript (see Figure 1). We construe temporal proximity for both preceding and following target utterances because memory models suggest that both forward and backward integration over time may arise across dynamic experiences (Howard & Kahana, 2002).

The average distance between noun instances was 28.34 utterances (95% CI [27.81, 28.81]), and 28.57 utterances (95% CI [28.21, 28.92]) for verbs. In order to compute the distance between repetitions, we needed at least two instances of a given word within a transcript. Therefore, in analyses containing this measure, we excluded noun instances that appeared only once in the transcribed interaction (N = 14,287; 13.3%). Similarly, for verb analyses, we excluded 12,026 (7.6%) verb instances for the same reason. For all other analyses, we included all noun or verb instances, respectively.

##### Transcript-Level Measures.

For each transcript, we computed the proportion of word instances that were isolated (vs. multi-word). In addition, we computed the proportion of word instances that were part of a repetition cluster (see Figure 1), that is, if the target word occurred at least three times within six consecutive caregiver utterances (Onnis et al., 2008a, 2008b; Schwab et al., 2018). In order to reduce the skew of these proportion estimates, we took the log of the proportion (after adding a constant factor of 0.01 in order to avoid undefined log values for proportions of 0, and retain proportion values between 0 and 1), and z-scored these values.

##### Analyses.

In order to model the distance between a given instance of a word and its closest repetition in caregiver speech, we used a Poisson zero-inflated mixed effect model, to account for the dependent variable being a count (number of utterances) and having many values of zero (i.e., back-to-back repetitions). We included a fixed effect of isolation (vs. multi-word), coded as a categorical variable, with multiword utterances treated as the baseline condition, and random intercepts and slopes per word, speaker, and transcript, in order to account for the nested structure of the dataset.

### Results

#### Isolated (vs. Multi-Word) Word Instances Are Closer to Their Nearest Repetition.

**Figure 2.**
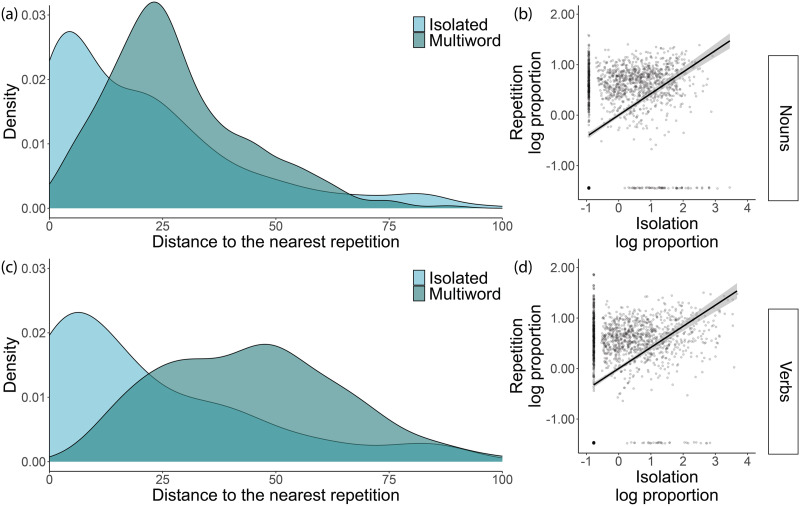
Panels (a) and (c) show the probability density function of repetition distances for isolated (in lighter blue) vs. multi-word (in darker blue) instances of the MB-CDI (a) nouns and (c) verbs in English caregiver speech in CHILDES. There is a higher density of close repetitions (e.g., less than 10 words) for word instances that are isolated. For visualization purposes, we are only displaying distances of fewer than 100 utterances. Panels (b) and (d) display the association between the proportion of word instances that are isolated (on the *x*-axis) and the proportion of word instances that are part of a repetition cluster (on the *y*-axis) for (b) nouns and (d) verbs. Each point represents a single transcript. The black line shows the linear relation between these two values, and the shaded interval around it represents a 95% confidence interval. In order to reduce the skew of the data, the units for both axes were converted to the log proportion of instances and z-scored. In order to avoid undefined log values and retain proportion values between 0 and 1, a constant factor of 0.01 was added to all values.

To assess the relation between isolation and repetition at the utterance level, we used a Poisson zero-inflated mixed effect model with a fixed effect of isolation (vs. multi-word) and random intercepts and slopes per word, speaker and transcript, predicting the number of utterances between a noun instance and its closest repetition. Isolation was negatively related to repetition distance for nouns (*β* = −1.32, *z* = −8.50, *p* < 0.001; Figure 2a), such that isolated instances of a noun were, on average, closer to another instance of the same noun, compared to multi-word instances (see Figure 2a). Isolated noun instances were, on average, 26.73 instances (95% CI [24.44, 29.01]) away from their closest repetition, whereas instances embedded in a multi-word utterance were approximately 28.43 utterances (95% CI [27.89, 28.96]) away from their closest repetition. In addition, 30.9% of all isolated noun instances were also part of a repetition cluster, and 23.7% of multiword instances were part of repetition clusters that did not contain an isolated instance of the noun (*χ*^2^ (1) = 52.37, *p* < 0.001). As one example (Valian, 1991), in back-to-back utterances, a parent said, “It’s an elephant,” “Elephant,” “Where’d you see an elephant?”. Similarly, in a different example, a parent said “Here, push,” “Push,” “You push on the button.”

We observed the same effects for verbs. Isolated instances of a verb were closer to other instances of the same verb (*β* = −1.46, *z* = −7.22, *p* < 0.001; Figure 2c). Isolated verbs were, on average, 18.53 utterances (95% CI [16.89, 20.17]) away from the closest repetition, whereas multi-word verb instances were 28.82 utterances (95% CI [28.45, 29.18]) away from the closest repetition. Similarly, 22.1% of isolated verb instances were part of repetition clusters, compared to 13.8% of multiword instances that were part of repetition clusters without an isolated instance of the verb (*χ*^2^ (1) = 167.97, *p* < 0.001).

#### Caregivers’ Use of Isolation and Repetition Are Correlated.

An exploratory analysis at the transcript level showed a correlation between the proportion of isolated and of repeated noun instances in a given transcript (*r* = 0.46, *p* < 0.001; Figure 2b). We found a similar correlation in the proportion of isolated and of repeated verb instances (*r* = 0.42, *p* < 0.001; Figure 2d). These results are consistent with the hypothesis that isolation and repetition are intertwined rather than independent in caregivers’ use of nouns and verbs. In a supplementary analysis, motivated by the decrease in caregiver use of repetition over the second year of life observed in prior studies (Schwab et al., 2018), we showed that the correlation between caregivers’ use of isolation and repetition decreased with age (Supplementary Figure 2), based on available data in CHILDES.

## STUDY 2: HOW DO ISOLATION, REPETITION, AND THEIR CO-OCCURRENCE MATTER FOR NOUN AND VERB LEARNING?

We hypothesized that the co-occurrence of repetition and isolation observed in Study 1 would facilitate child production. In Study 2, we predicted that children would produce nouns and verbs that occur more often in isolation-repetition clusters (i.e., clusters of repetitions of a word that also contain isolated instances) at an earlier age, even after controlling for their frequency of independently occurring in isolation and in repetition clusters.

### Method

#### Dataset.

We quantified caregiver input using the same North-American English CHILDES utterances as in Study 1. We assessed normative child production using the Wordbank dataset (wordbankr, version 1.0; Braginsky, 2023; Frank et al., 2017, 2021) of the productive vocabularies of 9,093 English-speaking toddlers between the ages of 16 and 30 months on the MB-CDI Words & Sentences form (Fenson, 2007).

#### Measures

##### Child Production.

Using Wordbank (Braginsky, 2023; Frank et al., 2017, 2021), we assessed child production in two ways. First, for the main analysis, we computed the age at which 50% of children produced each noun (normative age of acquisition) by fitting a logistic curve, using the fit_aoa function in wordbankr to the toddler productive vocabularies in Wordbank (Braginsky, 2023; Frank et al., 2017, 2021). Second, for an exploratory analysis, we computed the proportion of children producing each noun in 1-month bins between ages 16 and 30 months, for example, the proportion of children producing each noun at 9 months, at 10 months, and so on. We carried out exploratory replications of both analyses with verbs.

##### Utterance-Level Measures.

As in Study 1, word instances were tagged as *isolated* vs. *multi-word*. In addition, each word instance was marked as being part of a *repetition cluster* if it was part of a repetition sequence in which the target word occurred at least 3 times within 6 consecutive caregiver utterances. Child utterances were not counted in determining the bounds of a repetition cluster. Finally, word instances were marked as being in an *isolation-repetition cluster* if they were part of a repetition cluster and at least one of the target word instances within the repetition cluster was isolated.

##### Frequency in Caregiver Input.

For each word, the log frequency of occurring in isolation, in repetition clusters, and in isolation-repetition clusters was computed as the log of the total number of instances of the target word in each of these structures. A constant factor of 1 was added to all values, to avoid indeterminate log numbers, while keeping all numbers as integer counts. These estimates were z-scored for easier comparison of effects across the different structures.

We carried out two analytical approaches, one using an aggregate summary of language input and one considering developmental change over time. For the first analysis predicting age of acquisition, we quantified an aggregate summary of the log frequency of a word occurring in the corresponding structure in all corpora spanning the full age range (9 to 30 months). For example, the noun “hand” occurred 25 times in isolated utterances, 353 times as part of repetition clusters, and 17 times as part of isolation-repetition clusters in this aggregate dataset. Although including this broad age range means that some of the input would fall after the age of acquisition (e.g., the input from 24–30 months if the age of acquisition for a given noun is 24 months), we chose this bird’s eye view approach for two reasons: (1) it allowed us to estimate the frequency of isolation, repetition and their combination over the same amount of data for each word, and (2) it bypassed the uncertainty around when each individual child learned the word (relative to the normative age of acquisition, computed over a separate dataset).

Considering developmental change over time, in an exploratory analysis, we computed a cumulative version of the log frequency measure described above, summarizing caregiver input before the age at which production was measured, and used it to predict the proportion of children producing each word at different ages. This resulted in an input and production data point for each word at each age. For example, in order to predict child production at 16 months, we computed the log frequency of isolation, repetition clusters, and isolation-repetition clusters in caregiver input before 16 months; because our CHILDES data started at 9 months, this range was from 9 until 16 months. Similarly, to predict production at any age *a*, we assessed caregiver input to children between 9 and *a* months. This approach only characterized input received prior to the time when production was assessed. In order to account for the fact that the proportion of children producing any word increases with age, we centered this variable around the average proportion of children producing any word in the dataset for that age.

##### Analyses.

In order to model the age of acquisition of a given word as a function of its frequency in isolation, repetition, and isolation-repetition clusters (coded as continuous z-scores), we used a Gamma regression model to account for the fact that age of acquisition is a strictly positive number. When modeling the proportion of children producing a given word at a given age, we used a beta regression, reflecting that proportions are bounded between 0 and 1. To account for the skewed distribution of word frequencies, we logged and z-scored all frequencies and controlled for the overall log frequency of the word.

### Results

#### Isolation and Repetition Independently Predict Earlier Age of Acquisition.

First, in order to replicate past work suggesting that isolation and repetition independently predict earlier acquisition (e.g., Brent & Siskind, 2001), we created two Gamma regression models predicting the normative age of acquisition of each noun from its log frequency in repetition and isolation respectively, controlling for overall log frequency. Replicating prior findings, we found that greater frequency in isolation (*β* = −0.07, *t*(291) = −7.08, *p* < 0.001) and repetition (*β* = −0.15, *t*(291) = −7.91, *p* < 0.001) predicted earlier age of acquisition. For example, even though the words “bear” and “duck” had similar overall frequency in our data, the word “duck” appeared more often in isolation and had an earlier age of acquisition (Table 1). Similarly, even though the word “eye” was less frequent than “hand” in our data, it appeared more often in repetition clusters and was learned earlier (Table 1). We found similar results when we replicated this analysis with verbs (isolation: *β* = −0.03, *t*(97) = −4.026, *p* < 0.001; repetition: *β* = −0.06, *t*(97) = −3.35, *p* < 0.001). These patterns replicate prior findings that children learn words that occur more frequently in isolation or in repetition clusters earlier.

**Table 1.** Example words and their age of acquisition as a function of the proportion of their instances that arose in isolation-repetition clusters.

	**Age of Acquisition**	**Total Instances**	**Isolated**	**Repetition Cluster**	**Isolation-repetition Cluster**
eye	18	986	5.7%	30.4%	7.5%
hand	22	1661	1.5%	21.2%	1.0%
apple	19	644	20.8%	29.0%	9.3%
orange	23	828	17.2%	21.0%	7.6%
duck	18	1456	12.0%	36.9%	11.3%
bear	21	1224	5.1%	34.6%	5.3%
climb	26	242	2.9%	6.6%	2.1%
drop	27	242	0%	9.1%	0%

#### Isolation-Repetition Clusters Predict Earlier Age of Acquisition.

**Figure 3.**
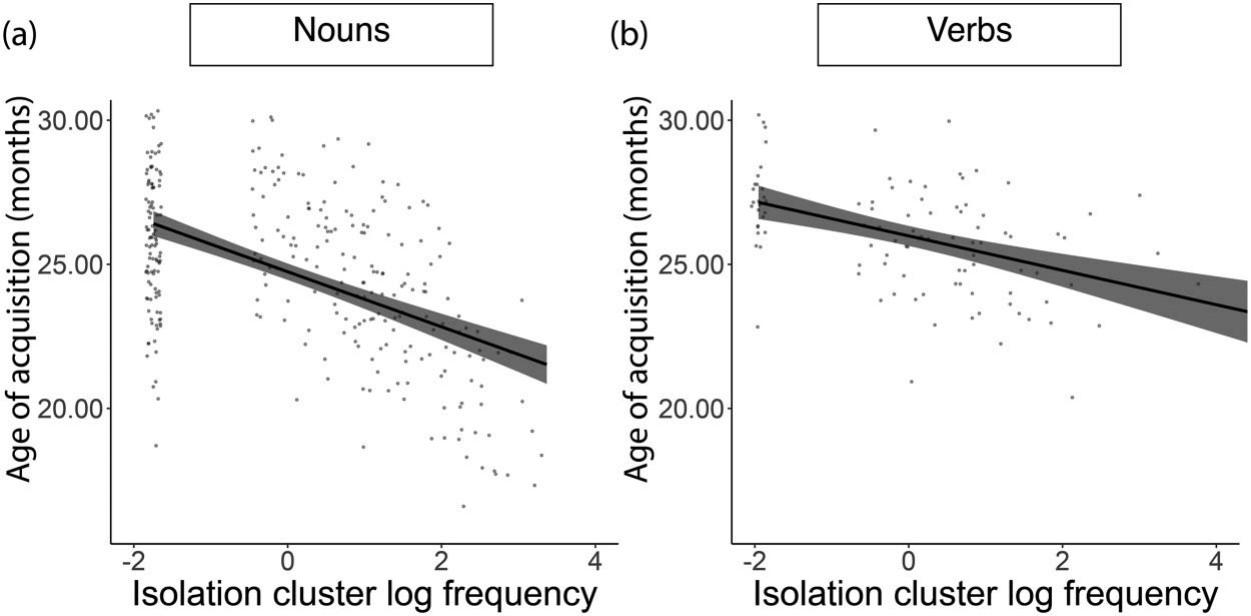
Panels (a) and (b) display each word type as a point, with the normative age of acquisition in months on the *y*-axis and the frequency of the instances for a given word being part of an isolation-repetition cluster for (a) nouns and (b) verbs. The *x*-axis units are a z-scored log of the number of instances of the word in isolation-repetition clusters (after adding a constant factor of 1 to all values to avoid undefined values for log of 0). Because repetition clusters were defined as having at least 3 repetitions, the lowest number of instances a noun could have in isolation-repetition clusters was 3. The gap in values on the *x*-axis represents the lack of nouns with 1 or 2 instances of an isolation-repetition cluster.

Next, we examined whether words that occurred more frequently in isolation-repetition clusters had an earlier age of acquisition (Figure 3). To do so, we used a Gamma regression model predicting the normative age of acquisition of each word from the log frequency of word instances occurring in isolation-repetition clusters, controlling for the log frequency of the word occurring in isolation and within repetition clusters. For nouns, the log frequency in isolation-repetition clusters was a significant predictor of age of acquisition (*β* = −0.03, *t*(290) = −2.23, *p* = 0.03), even when controlling for isolation and repetition. When including isolation-repetition clusters, the age of acquisition of nouns was no longer significantly associated with their log frequency in isolation (*β* = −0.02, *t*(290) = −1.42, *p* = 0.16) or repetition (*β* = −0.02, *t*(290) = −1.46, *p* = 0.14). Although isolation-repetition clusters were also a significant predictor of age of acquisition in verbs when controlling for overall frequency (*β* = −0.03, *t*(97) = −3.47, *p* < 0.001), this relation did not remain significant after controlling for the frequency in isolation and repetition (*β* = 0.003, *t*(96) = 0.21, *p* = 0.84). Isolation (*β* = −0.03, *t*(96) = −2.08, *p* = 0.04) and repetition (*β* = −0.02, *t*(96) = −2.70, *p* = 0.008), on the other hand, remained significant predictors of the age of acquisition of verbs when including isolation-repetition clusters. These results suggest that children more rapidly learn to produce nouns that occur more frequently in isolation-repetition clusters. One possibility, at least in the case of nouns, is that children learn words that are frequently isolated or repeated because they are part of a super-structure containing both isolation and repetition in close temporal proximity. For instance, even though the word “eye” was far less frequent than “hand” in our data, it appeared far more often in isolation-repetition clusters and was learned earlier (Table 1).

#### Isolation-Repetition Clusters Predict the Proportion of Children Producing a Noun.

We used a beta regression predicting the proportion of children producing a given target word at age *a* months from the log frequency (in ages 9 months to *a* months) of the target noun occurring in isolation-repetition clusters. For nouns, the log frequency in isolation-repetition clusters (*β* = 0.21, *t*(3865) = 4.45, *p* < 0.001) predicted the proportion of children producing the target noun at that age (centered and scaled by age), controlling for the log frequency of the noun occurring in isolation and repetition, and for the total frequency of the noun. As with age of acquisition, for verbs, isolation-repetition clusters predicted production only when controlling for overall frequency (*β* = 0.45, *t*(1541) = 12.28, *p* < 0.001), but not after controlling for the frequency in isolation and repetition (*β* = −0.07, *t*(1539) = −0.99, *p* = 0.32). These patterns suggest that when we consider only prior normative input, a larger proportion of children produce words that occur more frequently in isolation-repetition clusters; for nouns, this is the case even when we account for how frequently these nouns occur in isolation and in repetition clusters.

## DISCUSSION

What properties of language input matter for children’s learning? Recent lines of research, in which researchers appreciated aspects of a child’s experience that had previously eluded theorists, have revealed the payoff of pursuing evermore child-centered construals of early experience. For example, considering the child’s egocentric view or the multimodal nature of early experience has yielded important insights into the real-world signals that support learning (de Barbaro & Fausey, 2022; Karasik et al., 2011; Kosie & Lew-Williams, 2024; Kretch et al., 2023; Smith et al., 2011, 2015; Soska & Adolph, 2014; Soska et al., 2010; Yurovsky, Smith, et al., 2013). The current investigation expands this emphasis on children’s real-world experiences by centering the idea that children experience learning cues over time (across seconds to minutes during conversations). Prior research has decomposed the learning signal into independent features, such as word repetition on the one hand and isolation on the other hand, and assessed their effects on learning separately, without considering their co-occurrence. Applying a temporally grounded perspective to the domain of language input may change our theoretical predictions about which features of language input patterns support learning. For example, infants may track not only how often a word is repeated or isolated, but also benefit from rich and engaging moments that combine repetition and isolation. We discovered that two features of child-directed speech - isolation and repetition - are not, in fact, independent in children’s language input. Isolated (vs. multiword) instances of nouns were more likely to be part of repetition clusters, and isolation and repetition were correlated in caregiver speech. Further, our results suggest that the co-occurrence of isolation and repetition - a structure we referred to as isolation-repetition clusters - may be beneficial for word learning, as their frequency in children’s input was associated with words’ earlier age of acquisition.

Why might isolation and repetition co-occur? There are three related possibilities. First, isolation and repetition may co-occur because of discourse structure within early caregiver-child interactions. That is, unlike many lab-based experiments, children’s language input is grounded in social interactions, and the content and context of these interactions structure the timing of language experiences. Caregivers do not randomly jump between topics; instead, their talk about objects is typically clustered in short bursts (Slone et al., 2023). For example, children’s language input is organized around their daily routines, such that children encounter words for body parts during bath time and for fruits or vegetables during lunchtime (Custode & Tamis-LeMonda, 2020; Tamis-LeMonda et al., 2019). This may create moments when caregivers repeat and isolate the same word over short periods of time. Second, isolation and repetition in caregiver speech may be shaped by the goal of understanding and building upon what children say. In an earlier example from the Providence corpus (Demuth et al., 2006), within a repetition cluster for the word “fingers,” the child produced an unintelligible vocalization that the caregiver followed with the isolated utterance “Fingers” and the prompt “Can you say fingers?”. In this case, the caregiver may use the isolated instance to clarify what the child meant and promote common ground, or to facilitate the child’s comprehension of the communicative interaction. Finally, caregivers in a North American context may implicitly or explicitly use isolation and repetition together to support children’s word learning in the moment. In the “Fingers” example, the caregiver could have been intentionally scaffolding the child’s production of the target word, reflected in the isolated instance of the word being immediately followed by a prompt for the child to say the word. Future descriptive research modeling the situations when children encounter isolation and repetition at the same time can help us understand the function that this cue co-occurrence plays in everyday discourse, which in turn could inspire investigation of the temporal dynamics of many other language cues.

We found evidence that children show an earlier age of acquisition of words that are encountered more often in moments when caregivers combine isolation and repetition. Although we do not have specific answers, there are several reasons why isolation-repetition clusters may be related to earlier word learning. In the domain of language, there is evidence that multiple overlapping cues can be beneficial for learning and attention (Bahrick & Lickliter, 2000; Booth et al., 2008; Flom & Bahrick, 2007; Frank et al., 2009; Lew-Williams et al., 2019; Suarez-Rivera et al., 2019). For example, Suarez-Rivera et al. (2019) observed longer bouts of visual attention to an object when caregivers were both speaking about and touching the object. Similarly, combining touch and speech-based cues can help infants segment a speech stream into words (Lew-Williams et al., 2019). It is important to note that while isolation-repetition clusters predicted age of acquisition of nouns above and beyond isolation and repetition as individual features, this was not the case for action verbs. It is possible that there are different mechanisms through which these features support learning about actions vs. concrete objects (which comprise a larger portion of nouns on the MB-CDI). Future research with a broader set of word types can allow us to understand how different combinations of cues may support learning for different types of words.

Quantifying potential temporal co-occurrences of multiple cues could yield insights about the encoding, integration, and retrieval mechanisms that are likely to drive word learning. For example, a learner who hears “*Where’d you see the elephant?*” may more strongly encode the sentence-final word “*elephant*” after they had just heard the single-word utterance “*Elephant*,” compared to if they had just heard a more complex multi-word utterance mentioning the elephant or even other topics entirely (e.g., due to heightened attention during encoding; deBettencourt et al., 2018). That is, the impact of a given experience may depend in part on prior experiences. Similarly, upon hearing “*Where’d you see the elephant?*”, a learner may retrieve prior instances of the word elephant and strengthen those memory traces (Howard & Kahana, 2002). They may be especially likely to do so if those prior instances had been recently experienced and/or robustly encoded (via an isolated-word exposure or otherwise), thereby ensuring minimal decay. That is, the impact of any experience may be updated by subsequent experiences. Thus, to the extent that cues known to be helpful for early word learning (e.g., isolation and repetition) also arise together in time, their power may derive in part from their joint impact on memory dynamics as learners build knowledge.

Yet another potential explanation is that isolation and repetition may reflect rather than create moments that are good for learning. For example, if a child is engaged with or vocalizing toward a toy, their caregivers may be more likely to talk about it, repeat its name or its actions, and provide clear isolated tokens of relevant words – and possibly enrich the interaction with other social cues including touch, pointing, or directed gaze. This may be especially the case for words that the child is interested in and learns earlier. These cue co-occurrences, whether isolation-repetition clusters or otherwise, may be more likely to appear in the moments when caregivers follow up on children’s interests. A related possibility is that there may be properties of the word itself that result both in greater use of the word in isolation-repetition clusters and in earlier age of acquisition. For example, caregivers may be more likely to repeat simpler or shorter words, which may also be learned earlier because they are easier to articulate. Similarly, concrete objects may afford more opportunities for repeated labeling, and their labels may also be easier to learn.

In order to disentangle these possibilities, there is a need for future descriptive work with a larger scope of cues, as well as experimental work testing the effects of different temporal dynamics of cues. When specifically considering the effects of isolation-repetition clusters, it is important to note that they are not abundant in the input. On average, only 3.8% of noun instances and 1.9% of verb instances were part of isolation-repetition clusters. Although this percentage varied across words (as seen in Table 1), this generally rare dynamic carried unique power in predicting age of acquisition. Going forward, it will be important to understand how these rare yet high-quality learning moments interact with longer-term learning processes (Clerkin & Smith, 2022; Kucker et al., 2015).

### Limitations

This investigation comes with several key limitations. First, we assessed production and input in separate datasets. Specifically, we summarized thousands of child vocabularies in the Wordbank database (Braginsky, 2023; Frank et al., 2017, 2021) to measure when children produce different words on average, and then, using a separate database (CHILDES; Braginsky et al., 2022; MacWhinney, 2000b), we aggregated over many transcribed caregiver-child interactions to annotate the presence of isolation-repetition clusters. Because of this approach, we were not able to probe how a specific child’s productions relate to their input, or the sources of variability across children’s experiences and productions. Densely sampled longitudinal corpora with measures of production and input (e.g., SAYCam; Sullivan et al., 2021) can allow us to get at these questions. Second, we only examined the temporal dynamics of two cues in language input. In order to truly examine how children extract cues from their input, a larger-scale, multi-feature, data-driven approach is needed, combining linguistic, social, and situational features in time. Third, the CHILDES database does not capture the full range of contexts and routines in children’s life experiences. It is possible that different activities such as free play, meal time, and book reading afford different amounts of repetition or isolated words (see also Montag et al., 2018). Fourth, we only measured the temporal dynamics of caregivers’ use of isolation and repetition, without taking into account the role of children themselves, as well as their siblings. To more comprehensively understand how these and other features of speech are co-constructed, it will be important in future research to examine contributions from children – both in initiating and extending interactions that may support their own learning. Finally, our analyses only covered North American English, and the families in the CHILDES database are not a representative sample of families across the globe, or even of North American families. The co-occurrence of repetition and isolation may be particular to cultural practices surrounding play in North America. Future work should examine how caregivers’ use of linguistic cues varies across cultures, languages, and communities; the power of cue co-occurrence for children’s learning will likely depend on how they are used in their environment.

### Conclusion

Reducing children’s language input to individual cues *a priori* can sometimes obscure potentially relevant descriptions of structure that children experience. Isolation-repetition clusters are just one example of how incoming input may be experienced in time by children, and here, we have shown that these clusters are related to the age of acquisition of many English nouns. This investigation is a step toward examining the timing of children’s language experiences, with the goal of rethinking what constitutes a ‘cue.’ This shift toward rich, dynamic models of input promises to open up new directions into how children may learn from experience, including new hypotheses about individualized experience-dependent learning (e.g., Samuelson, 2021).

## ACKNOWLEDGMENTS

We thank Rose Maier Hartman for early discussions and preliminary analyses. Portions of this research were presented at the International Conference on Interdisciplinary Advances in Statistical Learning.

## FUNDING INFORMATION

This research was funded in part by a grant from the National Institute of Child Health and Human Development to CLW (R01 HD095912).

## DATA AVAILABILITY STATEMENT

The data used in this investigation are from two publically available datasets and can be found on the corresponding websites: CHILDES (https://childes.talkbank.org/), and Wordbank (https://wordbank.stanford.edu/).

## Supplementary Material


