# Supplementary material for: Word Repetition and Isolation are Intertwined in Children’s Early Language Experiences
Source: Open Mind (Camb). 2024 Nov 22;8:1330–47. doi: 10.1162/opmi_a_00172 (PMC11627589; doi:10.1162/opmi_a_00172)
Supplement: Supplementary file 1 [file opmi-08-1330-s001.docx]

## **Supplementary Materials**

| 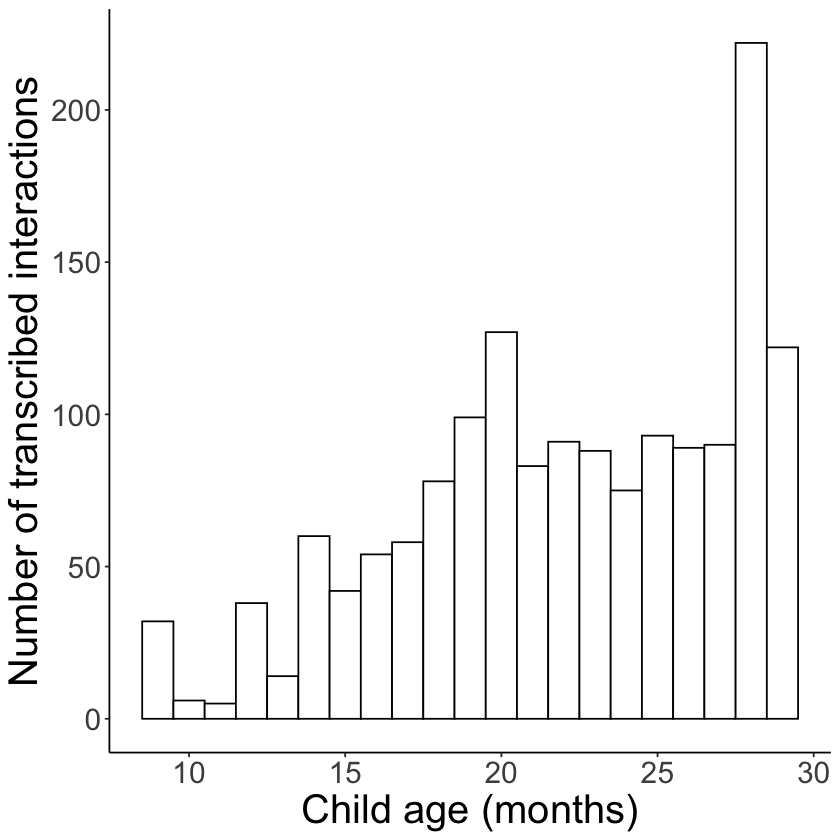 | **Supplementary Figure 1.** Histogram of the number of transcribed interactions in CHILDES across the analyzed age range. |
| --- | --- |

| (a) 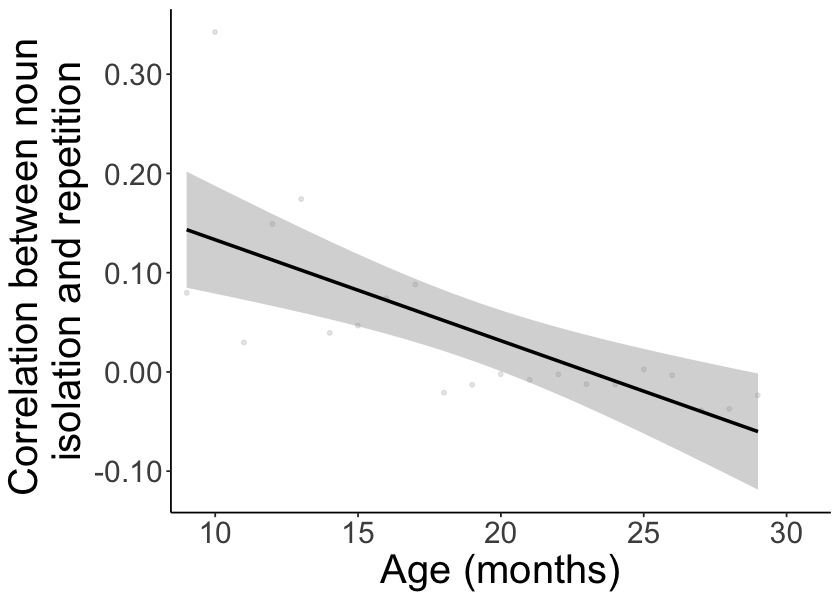 | (b) 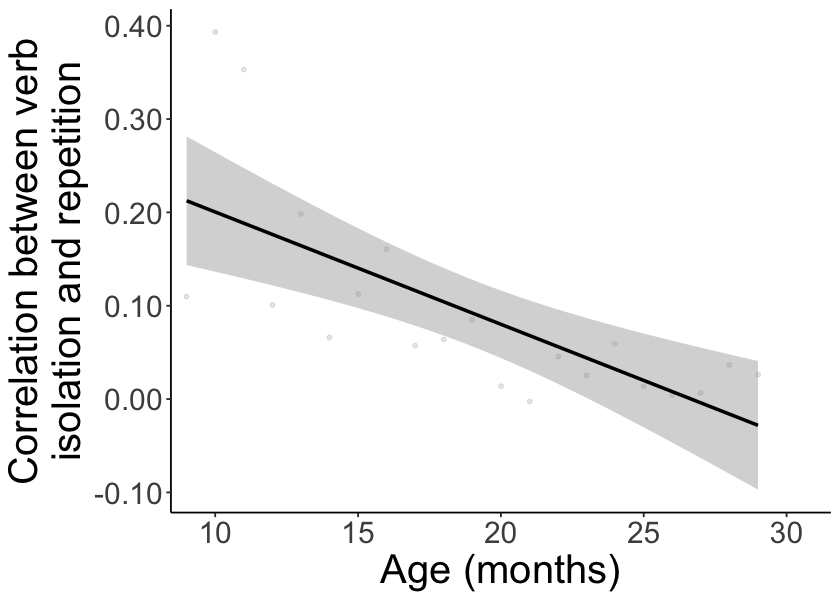 |
| --- | --- |
| **Supplementary Figure 2.** The correlation between caregivers’ usage of isolation and repetition decreased with age. The y-axis depicts the correlation between the proportion of instances of a given noun (a) or verb (b) token in a transcript that were isolated and the proportion of instances that were part of repetition clusters. The x-axis depicts the child’s age in months. | |
